# Estimation of optimal birth weights and gestational ages for twin births in Japan

**DOI:** 10.1186/1471-2458-6-45

**Published:** 2006-02-24

**Authors:** Noriko Kato, Tomohiro Matsuda

**Affiliations:** 1 Department of Education Training Technology and Development, National Institute of Public Health, 2-3-6 Minami, Wako-shi, Saitama, 351-0197, Japan; 2 Department of Epidemiology, National Institute of Public Health, 2-3-6 Minami, Wako-shi, Saitama, 351-0197, Japan

## Abstract

**Background:**

As multiple pregnancies show a higher incidence of complications than singletons and carry a higher perinatal risk, the calculation of birth weight – and gestational age (GA)-specific perinatal mortality rates (PMR) for multiple births is necessary in order to estimate the lowest PMR for these groups.

**Methods:**

Details of all reported twins (192,987 live births, 5,539 stillbirths and 1,830 early neonatal deaths) in Japan between 1990 and 1999 were analyzed and compared with singletons (10,021,275 live births, 63,972 fetal deaths and 16,862 early neonatal deaths) in the annual report of vital statistics of Japan. The fetal death rate (FDR) and PMR were calculated for each category of birth weight at 500-gram intervals and GA at four-week intervals. The FDR according to birth weight and GA category was calculated as fetal deaths/(fetal deaths + live births) × 1000. The perinatal mortality rate (PMR) according to birth weight and GA category, was calculated as (fetal deaths + early neonatal deaths)/(fetal deaths + live births) × 1000. Within each category, the lowest FDR and PMR were assigned with a relative risk (RR) of 1.0 as a reference and all other rates within each category were compared to this lowest rate.

**Results:**

The overall PMR per 1,000 births for singletons was 6.9, and the lowest PMR was 1.1 for birth weight (3.5–4.0 kg) and GA (40- weeks). For twins, the overall PMR per 1,000 births was 36.8, and the lowest PMR was 3.9 for birth weight (2.5–3.0 kg) and GA (36–39 weeks). At optimal birth weight and GA, the PMR was reduced to 15.9 percent for singletons, and 10.6 percent for twins, compared to the overall PMR. The risk of perinatal mortality was greater in twins than in singletons at the same deviation from the ideal category of each plurality.

**Conclusion:**

PMRs are potentially reduced by attaining the ideal birth weight and GA. More than 90 percent of mortality could be reduced by attaining the optimal GA and birth weight in twins by taking particular care to ensure appropriate pregnancy weight gain, as well as adequate control for obstetric complications.

## Background

Multiple birth rates are increasing world wide [1,2] as a consequence of the widespread introduction of assisted reproductive techniques. As multiple pregnancies involve more complications than singletons and have higher perinatal risk [3], thus the calculation of birth weight-gestational age (GA) specific fetal death rates (FDR) for multiple births is necessary in order to estimate the lowest FDR for these groups [4,5].

In Japan, while infertility therapy is in its introductory stages in comparison to the U.S., the twinning rate has continued to rise steadily since the middle of the 1970s [6,7]. In comparison with birth rates from 1951 to 1968, years in which multiple birth rates were stable, in 1997 birth rates increased 1.4 fold for twins, 4.7 fold for triplets, and 12.2 fold for quadruplets [7]. This increase in multiple birth rate gives rise to the need to assess and examine twin and triplet mortality rates. The purpose of this study is to estimate the birth weight and GA associated with the lowest perinatal death rate in contemporary Japan.

## Methods

In Japan, birth, death and stillbirth (GA more than 12 weeks) certificates are systematically stored on magnetic tape data files by the Ministry of Health, Labor and Welfare (MHLW). These certificates are filled in by obstetric clinicians or midwives following obstetric recording in the hospitals or clinics, and are filed in the city health department and changed into computerized files at the MHLW. This database contains information relating to sex, birth weight, birth length, GA, parity, ages of father and mother, and dates of birth and death. We used birth, infant death and stillbirth certificates of all multiple births reported between 1990 and 1999.

Among all live births and fetal deaths with a GA of 22 weeks and over, cases that correspond to the abnormal or missing data that occurred during data collection in the municipalities or during entry onto the magnetic tape database were excluded. This resulted in a 0.68 percent reduction of cases from the original population. Because birth and death certificates were not linked to each other, birth and death certificates of the same municipalities on the same day with the same job of the family were identified to be the same individuals. 96.5% certificates of early neonatal death were linked to birth certificates. The final study sample constituted 10,021,275 live births, 63,972 fetal deaths and 16,862 early neonatal deaths for singletons, which appeared in the table of the annual report from the MHLW, and 192,987 live births, 5,539 fetal deaths and 1,830 early neonatal deaths for twins from the magnetic tape database. For singletons, data on birth weight and GA-specific frequencies of births and perinatal deaths published in the MHLW annual reports were collected, and then compared with those relating to twins. Frequencies were categorized by 500 grams for birth weight and by four week intervals for GA.

The FDR of each plurality, according to the birth weight and GA category, was calculated as follows: fetal deaths/(fetal deaths + live births) × 1000. Similarly, the perinatal mortality rate (PMR) of each plurality, according to the birth weight and GA category, was calculated as follows: (fetal deaths + early neonatal deaths)/(fetal deaths + live births) × 1000.

For each plurality type, the lowest FDR and PMR were identified within the categories of birth weight and GA. Within each category, the lowest FDR and PMR were assigned with a relative risk (RR) of 1.0 as a reference and all other rates within each category were compared to this lowest rate, calculating RRs ± 95 percent confidence intervals (CIs). RR was also calculated by comparing the same birth weight and GA category for twins versus singletons.

## Results

For live twin births, the mean birth weight was 2,346 g and the mean GA was 37.14 weeks. The mean birth weight of stillborn twins was 747 g and the mean GA was 29.85 weeks.

The overall FDR/PMR per 1,000 births for singletons was 5.4/6.9, and the lowest FDR and PMR was 0.6/1.1 for birth weight and GA (3.5–4.0 kg and 40- weeks/3.0–3.5 kg and 36–39 weeks) (Table 1) [see Additional file 1]. For twins, the overall FDR/PMR per 1,000 births was 27.9/36.8, and lowest FDR/PMR was 2.7/3.9 for birth weight and GA (2.5–3.0 kg and 36–39 weeks) (Table 2) [see Additional file 2].

**Figure 1:**
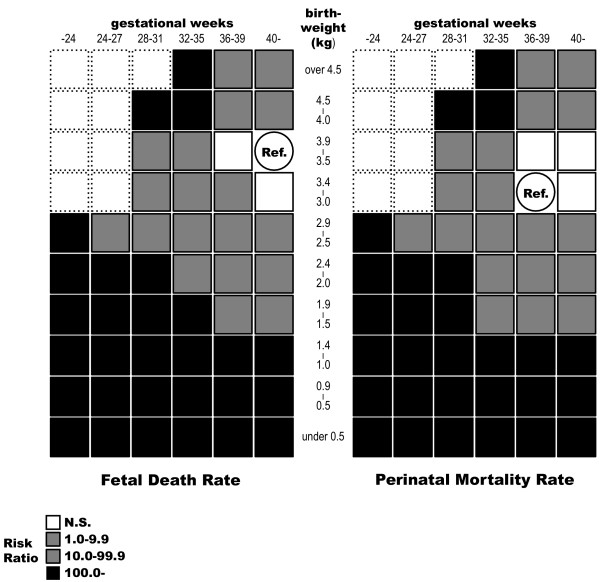
**Single/lowest single risk ratio**. The risk ratio for each birth weight and gestational week category is shown. Birth weight and gestational weeks category for the lowest mortality is a reference with a risk ratio of 1.0.

For singletons, the lowest FDR/PMR was found to be at 3.5–4.0 kg and 40- weeks/3.0–3.5 kg and 36–39 weeks, although the risk of fetal death was not significantly different at the neighbouring intervals (Figure 1). In gestations of less than 35 weeks, within the birth weight and GA categories, the highest risks for fetal and perinatal death were found among the lowest birth weights, showing a strong association between the intrauterine growth retardation and fetal/perinatal death.

**Figure 2:**
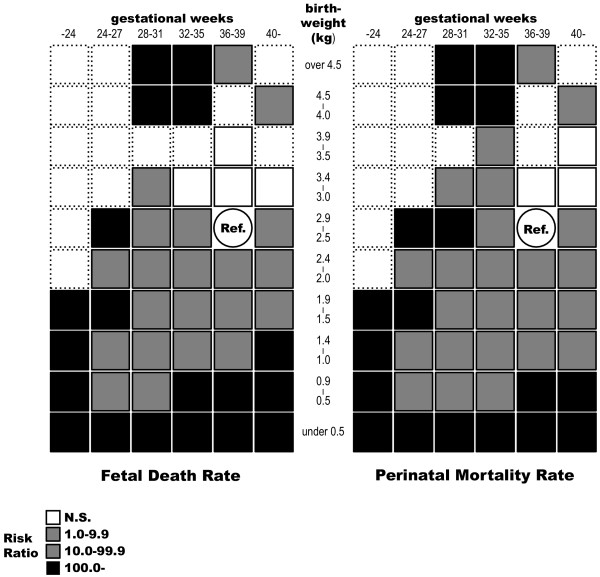
**Twin/lowest twin risk ratio**. The risk ratio for each birth weight and gestational week category is shown. Birth weight and gestational weeks category for the lowest mortality is a reference with a risk ratio of 1.0.

For twins, the lowest FDR/PMR was found at 2.5–2.9 kg at 36–39 weeks, although the risk of fetal death/perinatal mortality was not significantly different at the neighbouring intervals (Figure 2). The RR did not increase dramatically until the birth weight was reduced to 1.5–1.9 kg, and to 32–35 weeks for the gestational period. Beyond these ranges, the risk of fetal death increased as each category decreased, i.e. birth weight, GA or both, with significant differences. The increase in risk with which each category deviated from the ideal was less evident for twins than singletons. The overall RR for fetal death/perinatal mortality is higher for twins than for singletons. Among twins, intrauterine growth retardation as a risk factor was equal to that of immaturity, with a larger increase in risk across birth weights for a given GA than across GAs for a given birth weight. The negative effect of intrauterine growth retardation is more important in twins than in singletons with a higher elevation of RR at categories with higher birth weights and older GA.

Proportions of births included in the categories with moderate risk (RR<10.0) were 92.7 percent (singleton) and 90.0 percent (twins) for FDR, and 97.1 percent (singleton) and 89.3 percent (twins) for PMR.

**Figure 3:**
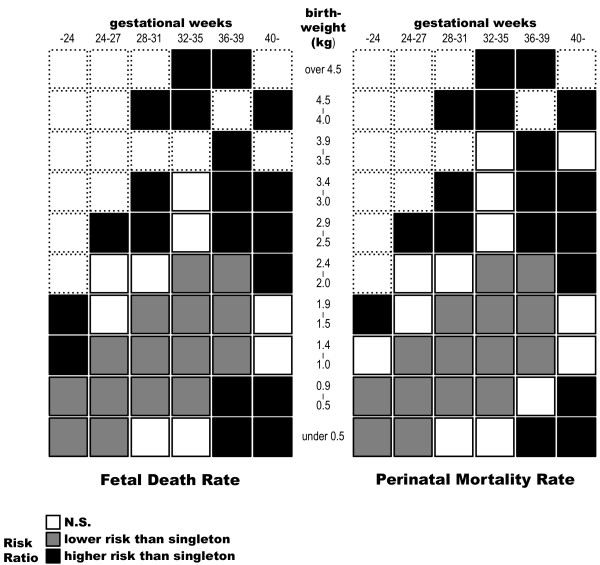
Twin/single risk ratio.

The category-specific comparisons of FDR/PMR for twins versus singletons is shown in Figure 3. Generally, at a birth weight in the category under 2.5 kg and GA in the category period below 36 weeks, twins had significantly lower category-specific risks for fetal death/perinatal mortality compared to singletons, and significantly higher risks at birth weights and GAs above these ranges.

## Discussion

The optimal birth weight and GA were evaluated for singletons and twins. Even with premature GA, adequate intrauterine growth is consistently associated with the substantial reduction in the risks of fetal death. For each plurality, PMR or FDR is the lowest at the optimal birth weight and GA, the deviation from which makes PMR or FDR higher. If the deviation is the same, PMR or FDR is said to rise higher among the triplets than among twins, and also higher among twins than among singletons [4]. Thus, the attainment of the ideal birth weight and GA are critical above all other factors in multiple pregnancies; however, these risks can be dramatically reduced by the attainment of ideal plurality-specific birth weight and GA. Fetal death and perinatal mortality in multiple births could be reduced by the attainment of optimal GA and birth weight. Adequate weight gain in pregnancy effectively prevents intrauterine growth retardation [8,9]. Moreover, adequate control for obstetric complications leads to the attainment of optimal GA and intrauterine growth. Specialized care for multiple gestations is reported to improve newborn outcomes and to reduce costs of neonatal care [10].

The statistical analysis in this study has confirmed that intrauterine growth retardation is associated with the risk of fetal and neonatal death, as well as prematurity. This can be seen clearly in the results for the category with relatively large GA and relatively small birth weights [4,5]. Previous reports point out that intrauterine growth retardation is associated with an increased risk of fetal and neonatal death [2,11-13]. Moreover, intrauterine growth retardation is associated with higher rates of morbidity among survivors [14]. Our study confirmed this tendency, observing that intrauterine retardation has a risk equal to that of immaturity.

The lengths of GA were filled in into birth/stillbirth certificates following obstetric records in the hospitals or clinics. Some of them were calculated from the last menstual period and others were identified using ultrasonic measurements. Although the assessment of GA with the last menstual period only may lead to over/under estimation, the proportions are so small that the overall conclusion would not be different, even if all the GA information were based on the ultrasonic measurements.

This analysis also confirmed that the FDR/PMR for twins is lower than for singletons in earlier GA or lower birth weight ranges. This finding has been reported previously [4,5]. In other words, the optimal GA for twins was earlier than for singletons and the optimal birth weight was lower. In this study, we aimed to compare mortality rates within each plurality and among pluralities through the absolute classification of birthweight and GA. Although categories of birth weight and GA that were used were not useful from a prognostic point of view, we should use them for comparison with singletons because they were the same as those published in MHLW annual reports concerning the mortality of singletons, which is the limitation of the present study.

These results imply that the singleton range of birth weight and GA maturity is actually at a level that is postmature for twins. In reference to biological evidence, it has been reported that fetal lung maturation occurs several weeks earlier in twins than in singletons [15]. The incidence of bronchopulmonary displasia in twins has been reported at lower rates than in singletons with birth weights lower than 1.5 kg or with GAs earlier than 32 weeks.

Overall, the results of this study are similar to the findings of Luke [4], whose study similarly reported the optimal birth weight and GA for twins (2.5–2.8 kg and 36–37 weeks). Luke [4] mentioned that the overall versus lowest fetal death rate per 1,000 births for twins 15.5 versus 3.3 at 2500 g – 2800 g and 36–37 weeks; and that FDR can be reduced by 75–80 percent with the attainment of birth weight and GA within a plurality-specific ideal range. Adopting this calculation in our database, FDR/PMR can be reduced by 84–90 percent with the attainment of the ideal birth weight and GA. Although similar results to the present study were discussed previously [4,5], the importance of the present study is that it clarified the optimal birth weight and GA in the Japanese population.

The detailed differences between Luke's and our study are probably due to ethnic differences in the study population or differences in neonatal care practices. In addition to the different observation period, Luke's study was based on the period 1983–88, while our study was conducted between 1990–99. Although the observation period of this study is more recent, the FDR in Japan was much higher (15.5 per 1,000 births in the United States against 27.9 in Japan). This reflects differences in the reporting of fetal deaths and early neonatal deaths between Japan and the US. In Japan, early neonatal deaths with life durations as short as a few hours tend to be classified as fetal deaths. Moreover, the sample size of this study was smaller than that of Luke's, particularly in relation to twins, although our observation period was longer. However, this is not due to differences in the exhaustibility of data collection, but is simply due to differences in the general population sizes in Japan and the US.

Recently, studies were performed with various approaches to deal with the issue about optimal birth weight and GA. Ananth et al. took medically indicated preterm delivery (labor induction and cesarean delivery) into consideration[16,17].

The methodology of the present study was that of a conventional epidemiological approach. Recent publications have analyzed the PMR of multiple births by alternative fetuses with a risk approach[18,19], where twin births had consistently higher mortality rates than singletons at all gestational ages. This method provides new insight into the perinatal epidemiology in multiple births, the adaptation of which would be the issue for further investigations.

## Conclusion

We have clarified that the optimal GA was earlier and the optimal birth weight was lower in multiple births in comparison to singletons. More than 80 percent of mortality could be reduced by the attainment of optimal GA and birth weight in multiple pregnancies by taking particular care about pregnancy weight gain. It is hoped that the results of this study can contribute to the improvement of medical care guidelines providing clinical care for multiple pregnancies.

## Competing interests

The author(s) declare that they have no competing interests.

## Authors' contributions

All authors have made a substantial contribution to the publication. Noriko Kato, the principal author, performed all the research practice, analysis and reduction. Tomohiro Matsuda, who belongs to the same institute as the principal author, collaborated with the principal author to analyze the data and to discuss on the results.

## Pre-publication history

The pre-publication history for this paper can be accessed here:



## Supplementary Material

Additional file 1 Click here for file

Additional file 2 Click here for file
